# Flight behaviour diverges more between seasonal forms than between species in *Pieris* butterflies

**DOI:** 10.1002/ece3.70012

**Published:** 2024-07-17

**Authors:** Irena Kleckova, Daniel Linke, Francisko De Moraes Rezende, Luca Rauscher, Camille Le Roy, Pável Matos‐Maraví

**Affiliations:** ^1^ Institute of Entomology, Biology Centre CAS (Czech Academy of Sciences) České Budějovice Czechia; ^2^ Department of Zoology, Faculty of Science University of South Bohemia České Budějovice Czechia; ^3^ Experimental Zoology Group Wageningen University Wageningen the Netherlands

**Keywords:** flight kinematics, Lepidoptera, morphometrics, polymorphism, trajectory tracking

## Abstract

In flying animals, wing morphology is typically assumed to influence flight behaviours. Whether seasonal polymorphism in butterfly morphology is linked to adaptive flight behaviour remains unresolved. Here, we compare the flight behaviours and wing morphologies of the spring and summer forms of two closely related butterfly species, *Pieris napi* and *P*. *rapae*. We first quantify three‐dimensional flight behaviour by reconstructing individual flight trajectories using stereoscopic high‐speed videography in an experimental outdoor cage. We then measure wing size and shape, which are characteristics assumed to influence flight behaviours in butterflies. We show that seasonal, but not interspecific, differences in flight behaviour might be associated with divergent forewing shapes. During spring, *Pieris* individuals are small and have elongated forewings, and generally fly at low speed and acceleration, while having a high flight curvature. On the contrary, summer individuals are larger and exhibit rounded forewings. They fly at high speed and acceleration, while having high turning acceleration and advance ratio. Our study provides one of the first quantitative pieces of evidence of different flight behaviours between seasonal forms of two *Pieris* butterfly species. We discuss the possibility that this co‐divergence in flight behaviour and morphology is an adaptation to distinct seasonal environments. Properly identifying the mechanisms underpinning such divergence, nonetheless, requires further investigations to disentangle the interacting effects of microhabitats, predator community, parasitoid pressure and behavioural differences between sexes.

## Graphical abstract

Our study focuses on quantifying the flight behaviours of two closely related *Pieris* white butterflies and their seasonal forms, using high‐speed videography. Our results suggest that there are no interspecific but seasonal differences in flight behaviours. Our study provides quantitative evidence of different flight behaviours between seasonal forms of *Pieris* butterflies, and these differences were better predicted by forewing shape and size, rather than other factors (e.g. species identity and ambient temperature).

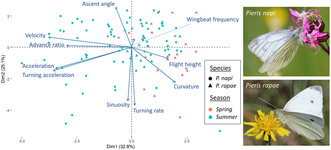

## 1 INTRODUCTION

Seasonal changes in the environment induce in some species the development of polymorphic forms exhibiting traits adapted to specific environmental conditions (Xue & Leibler, 2018). Such seasonal phenotypes are typically influenced by shifts in temperature (Nijhout, 2003), species interactions, such as the availability of feeding and breeding resources (Brakefield & Reitsma, 1991) or predators' abundance (Brakefield & Reitsma, 1991; Lyytinen et al., 2004). These adaptations are most common in regions with strong seasonality, where animals need to adjust their foraging, breeding or dispersal strategies for part of the year.

For flying insects living in temperate environments, wing shape and colouration are key polyphenic traits. Polyphenism of insect wings has been shown to play roles in thermoregulation (Kingsolver, 1985), interspecific signalling (Lyytinen et al., 2004) and powered flight (Harrison, 1980). However, while flight is the primary function of insect wings, little is known about its relation to wing morphology in the context of seasonal polyphenism. Differences in wing morphology are often associated with contrasted flight behaviours. For instance, wing loading (i.e. the ratio of body mass to wing area) has been associated with flight speed (Le Roy et al., 2019). Further, species with narrower and more elongated forewings (i.e. higher aspect ratio) might have a slower gliding and more energy‐efficient flight than species with more compact forewings, which might be better fit for manoeuvrable flight (Le Roy et al., 2019). However, less is known about how fine variations in wing shape within species affect flight performance (Breuker et al., 2007). Thus, our understanding of which specific wing traits influence distinct flight behaviours, including agility and manoeuvrability, remains unclear.

Seasonal flight behaviours may be adaptive. For example, searching flight behaviours of spring *Pieris* butterflies might be better suited to explore the scattered feeding and oviposition resources on their natal patch, while the powered flight of summer *Pieris* might be an adaptation for dispersal to novel localities (Fric et al., 2006; Shkurikhin & Oslina, 2016). In other butterfly groups, such as *Maniola jurtina* (Satyrinae), flight behaviours associated with nectaring were slower and more tortuous than movements associated with mate searching (Evans et al., 2020). Furthermore, seasonal changes in wing area might enhance dispersal ability, as quantified in migrating generations versus sedentary populations of *Danaus* butterflies (Tenger‐Trolander et al., 2023). Behavioural variation can also influence flight patterns in seasonal butterflies, as the preference in flight direction of migrating populations of *Pieris brassicae* seems to depend on the season of emergence (Spieth & Cordes, 2012). Thus, in the case of polyvoltine (i.e. species having several generations during a year) seasonal butterflies, the factors affecting intraspecific flight behaviours likely involve both morphological and behavioural adaptations (Spieth & Cordes, 2012; Tenger‐Trolander et al., 2023).

To assess whether seasonal changes in butterfly wing morphology affect their flight behaviours, we study two polyvoltine butterfly species, *Pieris napi* (Linnaeus, 1758) and *P*. *rapae* (Linnaeus, 1758). These two species are closely related (Okamura et al., 2019), co‐occur in Europe and are ecologically very similar (Tenger‐Trolander et al., 2023). They are known to differ in wing size and shape in the spring and summer (Fric et al., 2006; Shkurikhin & Oslina, 2016). The spring forms of both species might have a more manoeuvrable flight due to their elongated and pointed forewings (Shkurikhin & Oslina, 2016), whereas the summer forms might have improved dispersal capacity due to their larger wings and lower wing loading (Fric et al., 2006; Shkurikhin & Oslina, 2016). Furthermore, variation in habitat use is expected among both species and their seasonal forms (Friberg & Wiklund, 2019). While *P*. *napi* usually flies on forest edges and feeds on biennial and perennial plants, *P*. *rapae* explores temporary and degraded habitats with ephemeral plants (Ohsaki & Sato, 1999), including agricultural landscapes (Ryan et al., 2019). Seasonal differences in population dynamics driven by climate have also been reported for *P*. *napi* and *P*. *rapae* (Okamura et al., 2019; von Schmalensee et al., 2023); while *P*. *rapae* is more abundant during summer, *P*. *napi* has a higher overwintering survival than *P*. *rapae*, resulting in higher abundances during the spring (von Schmalensee et al., 2023).

Here, we quantify the flight behaviour of spring and summer *Pieris* using a stereoscopic high‐speed videography system in an outdoor cage environment. To test if divergent seasonality in wing morphology is associated with divergent flight behaviour, we compare the quantified flight characteristics (i) between *P*. *napi* and *P*. *rapae* and (ii) between seasonal forms of both species in the spring and summer. We expect larger differences in flight behaviour between seasonal *Pieris* forms than between species, which would point to an adaptation or seasonal plasticity in flight behaviour. Seasonal flight divergence may indeed benefit *Pieris* individuals by enhancing resource use in different environments (Fric et al., 2006; Shkurikhin & Oslina, 2016).

## 2 METHODS

### 2.1 Butterfly flight trajectories recording

The recordings took place from the end of May to mid‐August 2020. We caught *P*. *napi* and *P*. *rapae* in the field during the morning (~8:00–11:00) in the surroundings of České Budějovice, Czechia (48°59′28.3″ N, 14°26′29.6″ E). *Pieris* individuals captured between May and mid‐June represented the spring form, whereas the ones captured during July and August represented the summer form (von Schmalensee et al., 2023). A third generation of adults may fly during September and October during warm years (Benes et al., 2002), but these individuals have not been included in this study. Captured butterflies were stored in paper envelopes in a box with a wet napkin to avoid desiccation. To reduce specimen handling, we moved to the filming location immediately after field sampling, and the flight trajectories of individual butterflies were filmed.

Flight trajectories were filmed between 10:00 and 16:00 in an outdoor tunnel‐like cage (6 m × 2.5 m × 2.5 m, length × width × height) located in the lee of a building at the Biology Centre CAS campus. The cage's end, where we released the butterflies at the beginning of the recordings, was shaded to encourage them to fly towards the sunlit area of the cage. The individual butterflies were recorded for a maximum of 5 min. The tracks during which butterfly individuals fly spontaneously were included in the analyses.

Films were recorded using two GoPro HERO6 Black cameras mounted on tripods at a height of 80 cm, and positioned at perpendicular angle to each other, following a similar setup to that of Le Roy et al. (2021). The cameras recorded at 120 frames per second using the wide lens option with a resolution of 2560 × 1440 pixels. To uncover within‐individual variation, we filmed up to six flight trajectories per individual, depending on the fatigue of the butterfly individual and its ability to spontaneous flight. After the flight experiment, the butterflies were euthanized by freezing at −20°C, and later spread and mounted for morphological measurements. Ambient air temperatures during recording hours were obtained from a nearby (distance: 2 km) climatic station of the Czech Hydrometeorological Institute, České Budějovice‐Rožnov.

### 2.2 Quantifying flight parameters

To quantify the butterfly flight trajectories in three dimensions (3D), we used the software Argus v2.1 (Jackson et al., 2016) implemented in Python v. 3 (Van Rossum & Drake Jr., 2009). Because of the wide filming option, the coordinate systems from the camera videos were warped. We thus undistorted all videos using the Argus package *DWarp*. The camera setup was calibrated using the direct linear transformation technique (Theriault et al., 2014). For this, we used a 24‐cm ruler with Styrofoam balls fixed to both ends, which was moved across the cage. We then tracked the centre of the Styrofoam balls using the Argus package *Clicker*. The butterfly flight trajectories were manually tracked by digitising the positions of the butterfly thorax. Given its small size, we were not able to record the positions in certain video frames. We reconstructed the 3D coordinates per frame by merging the two synchronised 2D point trajectories and calibration coefficients using the Argus package *Wand*. The butterfly positions throughout the trajectory were then post‐processed using a linear Kalman filter in MATLAB (Muijres et al., 2014) resulting in smoothed positional coordinates as well as estimated missing positions. Additionally, the total number of wingbeats per butterfly trajectory was counted during the video processing.

The flight behaviour was described using 11 parameters for every individual butterfly. All the calculations were made using a custom‐written R script from Le Roy et al. (2021):

As general flight characteristics, we calculated (1) wingbeat frequency, as the average number of wingbeats per second, in Hz, (2) covered distance (in metres, m), which was calculated as the consecutive distance between all tracked 3D positions, (3) average flight height (m) and (4) mean velocity (in metre per second, m/s), and (5) mean acceleration (m/s^2^), which were calculated as the first and second temporal derivative of butterfly positions through time respectively.

As a measure of flight efficiency, we calculated (6) mean advance ratio, which describes the flapping efficiency and was estimated as the ratio of mean velocity to the wingbeat frequency (Ellington, 1984).

To describe individual turning abilities during flight, which encompass manoeuvrability and agility, we calculated (7) turning acceleration, which is a component of acceleration strictly attributable to changes in flight direction and (8) turning rate, which describes how quickly the butterfly turns, which was calculated as the angular change in the direction of subsequent velocity vectors in the 3D space.

To describe the flight trajectory shape, we calculated (9) sinuosity, which is the ratio of the straight distance between the starting and ending positions against the covered distance, (10) flight curvature, which is a measure of how sharply the butterfly turns (Jantzen & Eisner, 2008) and (11) ascent angle (degrees), calculated as the angle between the velocity vector and the horizontal plane.

### 2.3 Wing morphometrics

**FIGURE 1:**
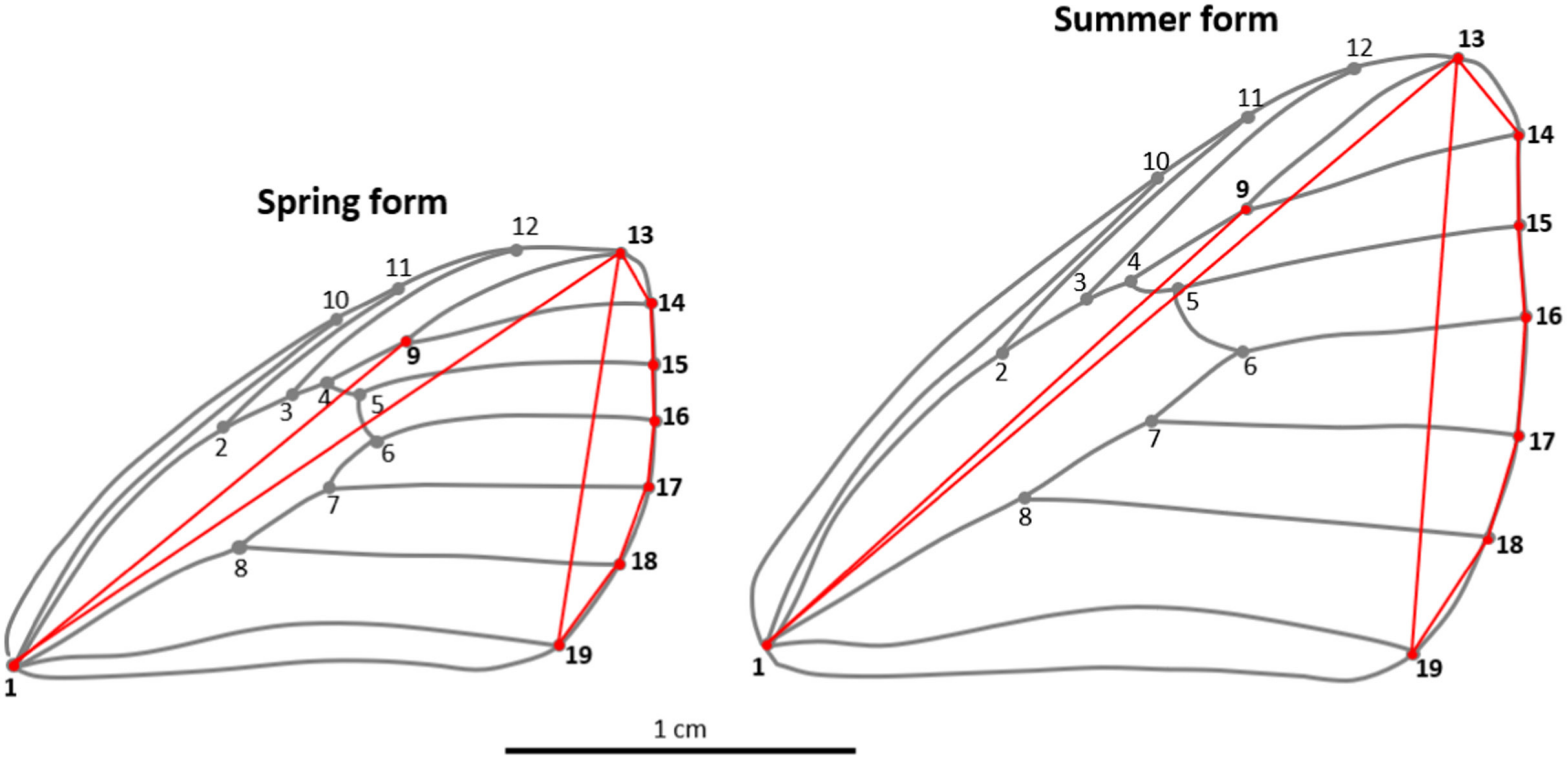
Wing morphology of *Pieris* white butterflies and their seasonal forms. Two indices of wing shape were calculated: the curvature of the forewing outer edge (the sum of distances around the outer edge from landmarks 13 to 19 divided by the straight distance between landmarks 13 and 19), and the relative length of the marginal region (the distance between landmark 1 and 9 divided by the straight distance between landmarks 1 and 13).

All butterflies were mounted and photographed with a millimetre scale, using a Canon EOS250D camera with an 18–55 mm EF‐S lens. We measured conventional wing morphometrics (Figure 1) using ImageJ v. 1.53 (Schneider et al., 2012) and, for the wing area calculations, we used GIMP (The GIMP Development Team, 2019). We measured the left wings for all calculations; if the left wings were damaged, the right wings were used instead. For all specimens, we calculated forewing length (in centimetres, cm), which is the distance between the wing base (landmark point 1; Figure 1) and the outer edge of vein on the landmark point 13 (Bai et al., 2015, Figure 1); forewing width (cm), which is the highest width perpendicular to the measured forewing length; aspect ratio, which characterises the relative elongation and narrowness of the forewings, and is calculated as the ratio of the forewing length to the width; forewing area (cm^2^); thoracic volume (cm^3^), which served as proxy to body mass, and was calculated as the cylindrical volume inferred from the thorax length and width; total wing area (cm^2^), measured as twice the forewing area (dorsal view) plus twice the hindwing area (ventral view), using the histogram function of GIMP; and wing loading, which is the ratio of thoracic volume (used as a proxy for body mass) to the total wing area.

In addition, we calculated two wing shape characteristics hypothesised to affect butterfly flight, which differ between species and between seasonal forms of *P*. *napi* and *P*. *rapae* (Shkurikhin & Oslina, 2016). First, the curvature of the forewing outer edge, which differ between the spring and the summer *Pieris* (Shkurikhin & Oslina, 2016), was calculated as the sum of distances around the terminal veins at the outer edge (landmark points 13, 14, 15, 16, 17, 18 and 19) divided by the straight distance between veins on landmark points 13 and 19 (Figure 1). A curvature of the forewing outer edge close to 1 represents the rounded forewings of summer *Pieris*, while an index higher than 1 depicts the elongated forewings of spring *Pieris* (Shkurikhin & Oslina, 2016). Second, we calculated the relative length of the marginal region, measured as the distance between landmarks 1 and 9 divided by the straight distance between landmarks 1 and 13 (Figure 1). This measure differs between the two study species (Shkurikhin & Oslina, 2016). The shape characteristics of one *P*. *napi* individual were not possible to calculate as its wing margins were damaged during handling after the video recordings; the specimen was excluded from the analyses of the effect of wing shape on flight behaviour.

We used the function *cor*.*test* of the R v. 4.2.1 (R Core Team, 2022) package corrplot (Wei & Simko, 2021) to test for possible pairwise correlations between the measured morphological variables (i.e. forewing area, aspect ratio, wing loading, curvature of the forewing outer edge and the relative length of the marginal region). Further, we used the base R function *aov* to test whether such morphological variables differ among species and seasonal forms, while accounting for the interactions between these factors.

### 2.4 Interspecific and seasonal differences in flight behaviour

We compared the differences in flight behaviour between species (*P*. *napi* and *P*. *rapae*) and between their seasonal forms, spring (May to mid‐June) and summer (July and August). First, the measured flight characteristics were reduced in dimensionality and visualised using principal component analysis (PCA). The function *imputePCA* of the R package missMDA (Josse & Husson, 2016) was used to estimate missing values of the average wingbeat frequency and advance ratio, in four 3D trajectories where wingbeats were difficult to score. To visualise possible interspecific and seasonal differentiation in flight behaviour, we used the function *fviz_pca* of the R package factoextra (Kassambara & Mundt, 2020).

Second, the factors explaining differences in flight behaviour among species and their seasonal forms were tested using linear models with mixed effects (LMMs), as implemented in the R package lme4 (Bates et al., 2015) via the function *lmer*. The response variables were the log‐transformed flight characteristics (1–11), except of (2) the covered distance, which was included as a fixed factor because it was used to derive the other flight characteristics. The response variables were fitted in separate models, and we had species, seasonal form and sex as predictor variables. As we recorded multiple trajectories per individual, specimen identity was included as a random factor. Then, post‐hoc, pairwise comparisons were performed for flight characteristics that were significantly different among species, seasonal forms or sexes in the LMM analyses. We used post‐hoc Tukey tests implemented in the R package emmeans (Lenth, 2022) to estimate marginal (*emmeans*) values of the LMMs for the evaluated flight characteristics for each species and seasonal form. In separate models, we first tested the effect of ambient temperature on flight characteristics in a LMM with species and seasonal forms as fixed factors and individual as random factor. Second, we tested for flight differences using simplified LMM with mean ambient temperature for each recording day as explanatory variable and butterfly individual as random factor.

### 2.5 Associations of wing morphologies with flight behaviour

We additionally tested the effect of morphology on flight while excluding species and seasonal form to focus on how morphological traits of recorded individuals affect each flight characteristics. For this, we used separate LMMs with each measured flight characteristic as the response variable. As explanatory variables in every LMM model, we used the log‐transformed forewing area, aspect ratio, wing loading, curvature of the forewing outer edge and the relative length of the marginal region. The log‐transformed (2) covered distance and sex were included as fixed effects, and specimen identity as a random factor. To test the effect sizes of the morphological variables on flight characteristics, we calculated Cohen's *d*, which was computed as the ratio of the estimated coefficient of the effect of a morphological variable to its standard error, providing a standardised measure of effect size. Next, we performed stepwise backward selection of the explanatory morphological variables using the function *step* of the R package lmerTest (Kuznetsova et al., 2017). This procedure aims to simplify the model by removing non‐significant predictors and selecting the most important morphological variable determining a specific flight characteristic. As an alternative approach to stepwise backward selection, we used dredge analysis using the R package MuMIn (Bartoń, 2009), which applies automated model selection based on Akaike information criterion. The algorithm considers all possible combinations of predictors to evaluate and rank a comprehensive set of candidate models formed from a global model.

## 3 RESULTS

### 3.1 Interspecific and seasonal differences in morphology

Morphological variables were uncorrelated with each other, except for a significant correlation between forewing area and aspect ratio (correlation coefficient −0.4012, *F*_29_ = 5.567, *p* = .028, Table S1). The differences in wing morphology between the two *Pieris* species were lower than between their seasonal forms, regardless of species (Table 1). The two *Pieris* species differed only in the relative length of marginal region (*F*_1,23_ = 9.111, *p* = .006), as *P*. *napi* had a longer marginal region than *P*. *rapae* (Figure 1 and Table 1). *Pieris napi* and *P*. *rapae* did not significantly differ in their forewing area (*F*_1,24_ = 0.0004, *p* = .863), aspect ratio (*F*_1,24_ = 0.007, *p* = .936), wing loading (*F*_1,24_ = 0.130, *p* = .722) nor the curvature of the forewing outer edge (*F*_1,24_ = 1.037, *p* = .319).

**TABLE 1:** Overview table summarising the number of studied male and female individuals of *Pieris napi* and *P*. *rapae*, and the *Pieris* seasonal forms (spring/summer), the number of flight tracks recorded, and the mean values with standard error (±SE) of their forewing area, wing loading (the ratio of thoracic volume to the total wing area), aspect ratio (the ratio of the forewing length to the width), curvature of the forewing outer edge and the relative length of marginal region.

	*N*	Spring/summer	No. of tracks	Forewing area (mm^2^)	Wing loading (mm^3^/mm^2^)	Aspect ratio	Outer edge	Marginal region
Males								
*Pieris napi*	14	3/11	51	248 (8.93)	0.0223 (0.00182)	1.75 (0.015)	1.09 (0.008)	0.72 (0.008)
*Pieris rapae*	12	5/7	38	242 (9.54)	0.0214 (0.00203)	1.74 (0.02)	1.09 (0.009)	0.68 (0.007)
Spring	8		22	225.6 (8.29)	0.0249 (0.00242)	1.77 (0.02)	1.11 (0.012)	0.70 (0.013)
Summer	18		67	256.3 (7.93)	0.0209 (0.00156)	1.73 (0.01)	1.080 (0.006)	0.71 (0.007)
Females								
*Pieris napi*	3	1/2	12	212 (29.1)	0.0164 (0.0022)	1.74 (0.055)	1.10 (0.020)	0.70 (0.010)
*Pieris rapae*	2	0/2	5	243 (22.4)	0.0160 (0.00104)	1.80 (0.015)	1.06 (0.012)	0.69 (0.033)
Spring	1		4	154 (NA)	0.0021 (NA)	1.85 (NA)	1.135 (NA)	0.70 (NA)
Summer	4		13	242 (9.2)	0.0015 (0.00008)	1.74 (0.035)	1.07 (0.006)	0.69 (0.016)

*Note*: *Pieris* species significantly differed only in the relative length of marginal region, whereas their seasonal forms differed in the other morphological measures. Generally, the spring forms are smaller with elongated forewings compared to the summer forms.

Seasonal forms differed in the size and shape of forewings (Table 1). For both species, spring individuals were smaller than summer ones. Spring *Pieris* had significantly lower forewing area (*F*_1,24_ = 9.934, *p* = .004) than summer *Pieris*. Although only marginally significant, wing loading (*F*_1,24_ = 3.447, *p* = .076) and aspect ratio (*F*_1,24_ = 3.956, *p* = .058) tended to be higher in the spring forms (Table 1). The higher aspect ratio values describes longer and slender wings, whereas the higher wing loading describes lower wing area in relation to body volume. The curvature of the forewing outer edge was significantly higher in spring *Pieris* (*F*_1,23_ = 9.766, *p* = .005), meaning that the spring generation had more elongated forewings, whereas the curvature of the forewing outer edge was closer to 1 in summer *Pieris*, meaning that the summer form had more rounded forewings (Figure 1). The relative length of the forewing marginal region (*F*_1,23_ = 0.045, *p* = .834) did not differ between spring and summer forms. We have not detected significant differences in morphological traits between sexes.

### 3.2 Interspecific and seasonal differences in flight

**FIGURE 2:**
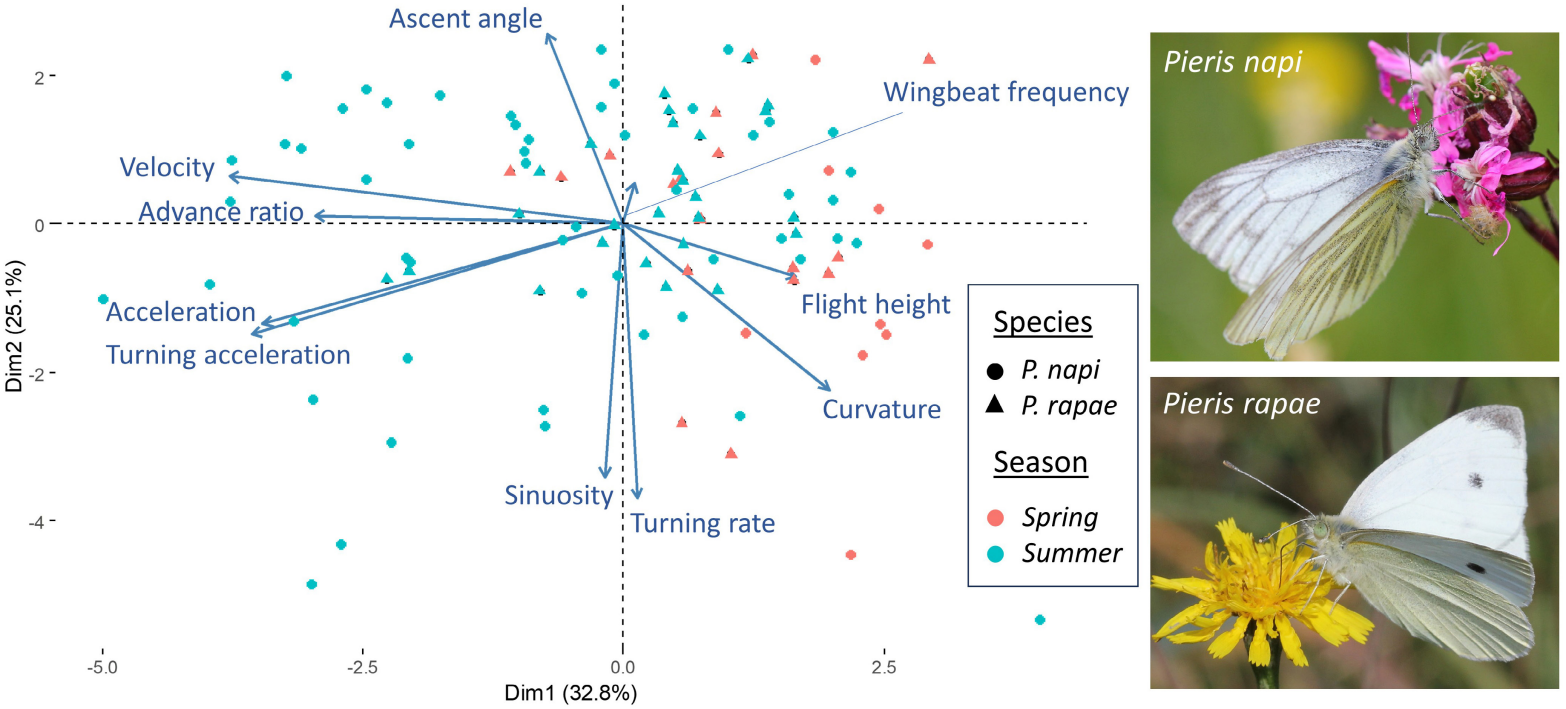
Seasonal forms but not species of *Pieris* butterflies differed in their flight characteristics. First and second axes of the PCA analysis of flight characteristics of the two closely related and sympatric *P*. *napi* and *P*. *rapae*, and their respective seasonal forms, spring and summer. Each point represents an individual flight trajectory (photos by Zdeněk Hanč).

We quantified 106 flight trajectories of 31 butterfly individuals: 14 *P*. *rapae* and 17 *P*. *napi* (Table 1). In the PCA performed on the set of flight parameters, the first PC axis (32.8% variance explained) was associated with higher velocity, acceleration, advance ratio and turning acceleration, and with lower flight height and flight curvature. The second PC axis (20.63% explained variance) was positively associated with ascent angle and negatively with mean turning rate and flight sinuosity. The PCA space did not reveal substantial differences between individuals of *P*. *napi* and *P*. *rapae*, as both had large variation in the measured flight parameters (Figure 2). Conversely, the flight behaviours of the spring and summer forms clustered separately, although higher variability in flight characteristics was present in summer individuals. Spring individuals had lower advance ratio, velocity, acceleration and turning acceleration, but higher flight curvature and flight height compared to the summer form.

Based on the results of the LMMs, the two *Pieris* species did not significantly differ in any of the measured flight parameters (Table 2). Further, males and females did not differ significantly in their tested flight characteristics, though this result should be interpreted cautiously due to the low number of recorded females (Table 1). However, while non‐significant, males tended to have curvier flight trajectories than females (*F* = 3.413, df = 30.06, *p* = .075); the mean flight curvature recorded in males was 3.15 (±0.520) and in females 1.85 (±0.416). In contrast, the seasonal forms differed significantly in their flight behaviours (Table 2), in agreement with the clustering pattern highlighted by the PCA; the spring individuals had lower advance ratio (*p* = .0215), velocity (*p* = .0064), acceleration (*p* = .029) and turning acceleration (*p* = .0045), but higher flight curvature (*p* = .0005) than summer individuals. The range of air temperatures recorded during the flight experiment was 14–36°C, with a mean value of 19 (±0.3)°C. We did not detect any significant effect of the air temperature on the measured flight parameters (Table S2).

**TABLE 2:** In an outdoor cage experiment *Pieris napi* and *P*. *rapae* butterfly species did not differ in flight behaviour, but their seasonal forms did.

Flight characteristic	Species effect			Seasonal form effect			Emmeans (±SE)	
	*F*	Dendf	*p*‐Value	*F*	Dendf	*p*‐Value	Spring (*N* = 26)	Summer (*N* = 80)
Wingbeat frequency	0.393	30.111	.53	0.532	32.548	.47	–	–
Flight height	0.002	28.692	.97	0.925	30.283	.34	–	–
Velocity*	0.006	27.087	.94	**10.928**	**28.864**	.002*	**1.2 (0.124)**	**1.7 (0.118)**
Acceleration*	0.008	25.561	.93	**5.001**	**27.238**	.03*	**2.31 (0.400)**	**3.43 (0.398)**
Advance ratio*	0.186	27.886	.67	**8.651**	**30.614**	.006*	**0.10 (0.013)**	**0.14 (0.012)**
Turning acceleration*	0.046	25.746	.83	**10.438**	**27.538**	.003*	**1.30 (0.232)**	**2.34 (0.278)**
Turning rate	0.002	28.751	.96	0.115	30.766	.737	–	–
Sinuosity	1.314	33.226	.26	0.762	35.440	.389	–	–
Curvature*	0.014	31.372	.91	**13.46**	**33.501**	<.001*	**3.80 (0.927)**	**1.53 (0.249)**
Ascent angle	0.334	28.215	.57	0.911	30.494	.35	–	–

*Note*: Comparison of interspecific differences in the flight characteristics of *P*. *napi* (17 individuals, and 63 trajectories) and *P*. *rapae* (14 individuals, and 43 trajectories), and between their seasonal forms, spring (9 individuals, and 26 trajectories) and summer (22 individuals, and 80 trajectories). For each *Pieris* individual, flight characteristics were inferred for several trajectories of differing length, thus, the linear mixed models (LMMs) included butterfly individual as a random factor, and the log‐transformed (2) covered distance and sex as fixed factors. For the flight characteristics that significantly differed among seasonal forms, the marginal emmeans of the LMM were calculated. Significant relationships are highlighted *in bold and with an asterisk*.

### 3.3 Relationships between wing morphology and flight behaviour

Flight characteristics were affected by forewing area and the curvature of the forewing outer edge, but not by aspect ratio, wing loading and relative length of marginal region. Full LMMs testing the effect of all the morphological variables on a flight characteristic revealed a positive effect of forewing area on flight advance ratio and a positive effect of the curvature of the forewing edge (i.e. describing the shape of *Pieris* forewings) on flight curvature (Table 3). In agreement with these findings, the stepwise backward selection indicated that the curvature of the forewing outer edge had large effects on flight curvature (slope = 11.84, SE = 4.35, *F* = 7.43, df 32.11, *p* = .01) and velocity (slope = −4.26, SE = 1.81, *F* = 5.53, df 31.24, *p* = .03). The larger forewing area found in summer *Pieris*, on the other hand, correlated with higher advance ratio (slope = 1.15, SE = 0.42, *F* = 3.36, df 31.45, *p* = .01). Similarly, the dredge analysis confirmed the relative importance of the predictor variables found by stepwise backward selection (per‐variable sum of model weights plots in Figure S1), highlighting the opposing effect of the curvature of the forewing outer edge on flight velocity (slope = −4.20, SE = 1.94, *F* = 4.67, df = 28.51, *p* = .04), and a large positive effect on flight curvature (slope = 14.54, SE = 4.50, *F* = 10.4631, df = 25.866, *p* = .003), whereas the larger the forewing area, the higher the flight advance ratio is (slope = 0.90, SE = 0.44, *F* = 4.23, df = 26.288, *p* = .05). Although we have detected a significantly higher turning acceleration (measure of turning ability) in summer *Pieris* (Table 2), we did not find any measured wing morphology trait predicting such a difference. Contrary to previous assumptions, we have not detected any significant effect of wing loading on any of the measured flight parameters between seasonal forms in *P*. *napi* and *P*. *rapae*. However, we remain cautious about this result because body mass was derived from estimates of thoracic volume and not from body mass.

**TABLE 3:** Flight performance of seasonal *Pieris* butterflies was affected by forewing area and the curvature of the forewing outer edge, but not by aspect ratio, wing loading and relative length of forewing marginal region.

Flight characteristic	Trait	Slope	SE	Cohen's *d*	*F*	Df	*p*‐value
Wingbeat frequency	Forewing area	−0.37	0.39	−0.96	0.92	23.71	.35
	Aspect ratio	1.66	1.62	1.03	1.05	24.24	.32
	Wing loading	0.04	0.18	0.24	0.06	22.67	.81
	Forewing edge	−0.32	1.85	−0.17	0.03	24.63	.86
	Marginal region	−0.12	1.73	−0.07	0.00	24.35	.95
Flight height	Forewing area	0.02	0.19	0.11	0.01	22.70	.91
	Aspect ratio	−0.73	0.79	−0.91	0.84	23.51	.37
	Wing loading	0.04	0.09	0.45	0.20	22.42	.66
	Forewing edge	0.98	0.89	1.09	1.20	23.44	.28
	Marginal region	0.48	0.84	0.57	0.33	23.02	.57
Velocity	Forewing area	0.75	0.42	1.78	3.18	23.48	.09
	Aspect ratio	−0.03	1.78	−0.02	0.00	24.48	.99
	Wing loading	0.12	0.19	0.65	0.42	22.95	.52
	Forewing edge	−2.99	2.00	−1.50	2.25	24.44	.15
	Marginal region	−0.24	1.87	−0.13	0.02	24.14	.90
Acceleration	Forewing area	1.10	0.69	1.60	2.56	22.79	.12
	Aspect ratio	−0.79	2.91	−0.27	0.07	23.71	.79
	Wing loading	0.17	0.32	0.54	0.29	22.36	.59
	Forewing edge	−2.57	3.28	−0.78	0.62	23.66	.44
	Marginal region	1.41	3.07	0.46	0.21	23.31	.65
Advance ratio	**Forewing area**	**1.04**	**0.46**	**2.25**	**5.06**	**21.67**	**.04**
	Aspect ratio	−1.91	1.94	−0.99	0.97	21.99	.33
	Wing loading	0.10	0.21	0.48	0.24	20.04	.63
	Forewing edge	−2.86	2.21	−1.30	1.68	22.34	.21
	Marginal region	−0.26	2.07	−0.12	0.02	22.41	.90
Turning acceleration	Forewing area	0.77	0.76	1.01	1.01	23.21	.32
	Aspect ratio	−3.01	3.21	−0.94	0.88	24.20	.36
	Wing loading	0.22	0.35	0.63	0.39	22.66	.54
	Forewing edge	−2.69	3.62	−0.74	0.55	24.16	.46
	Marginal region	3.83	3.39	1.13	1.28	23.88	.27
Turning rate	Forewing area	0.06	0.51	0.11	0.01	21.95	.91
	Aspect ratio	−2.21	2.19	−1.01	1.02	23.07	.32
	Wing loading	0.04	0.23	0.18	0.03	20.59	.86
	Forewing edge	2.22	2.46	0.90	0.82	23.37	.38
	Marginal region	3.02	2.31	1.31	1.72	23.83	.20
Sinuosity	Forewing area	−0.10	0.24	−0.42	0.18	25.06	.68
	Aspect ratio	−0.56	1.03	−0.54	0.30	26.27	.59
	Wing loading	−0.05	0.11	−0.46	0.21	23.54	.65
	Forewing edge	1.20	1.16	1.04	1.08	26.62	.31
	Marginal region	1.11	1.08	1.02	1.04	27.15	.32
Curvature	Forewing area	−0.99	1.01	−0.98	0.95	23.89	.34
	Aspect ratio	−1.16	4.28	−0.27	0.07	25.06	.79
	Wing loading	−0.16	0.46	−0.34	0.12	22.85	.73
	**Forewing edge**	**10.28**	**4.81**	**2.14**	**4.56**	**25.16**	**.04**
	Marginal region	1.20	4.51	0.27	0.07	25.27	.79
Ascent angle	Forewing area	0.18	0.57	0.31	0.10	22.35	.76
	Aspect ratio	0.24	2.39	0.10	0.01	23.04	.92
	Wing loading	0.26	0.26	1.00	1.00	20.79	.33
	Forewing edge	1.06	2.78	0.38	0.15	21.30	.71
	Marginal region	1.14	2.57	0.44	0.20	21.28	.66

*Note*: Linear models with mixed effects (LMM) testing the effects of morphological variables on each flight characteristic of *Pieris napi* and *P*. *rapae*. Log‐transformed forewing area, aspect ratio, wing loading, curvature of the forewing outer edge and the relative length of the forewing marginal region were used as explanatory morphological variables. The log‐transformed covered distance and sex were included as fixed effects and specimen identity as a random factor. The effect sizes were described by Cohen's d for individual explanatory variables included in one model with a flight characteristic as a response; a value of Cohen's |*d*| <0.2 indicates small effect size, |*d*| around 0.5 indicates medium effect size and |*d*| > 0.8 indicates strong effect of a morphological variable on a flight characteristic. The bold terms highlight the significant effect of a morphological variable on the flight characteristic (*p*‐value <.05).

## 4 DISCUSSION

In this study, we used stereoscopic high‐speed videography to quantify and compare the flight behaviours of two closely related *Pieris* species and their seasonal forms in an outdoor cage experiment. We hypothesised that *P*. *napi*, inhabiting forest edges, may fly with higher sinuosity and manoeuvrability compared to a potentially straighter flight in *P*. *rapae*, which is mainly found in agricultural open landscapes. However, this was not supported by our data, suggesting that flight adaptation to slightly different habitats use has not evolved in the studied species, as reported for other butterfly groups (e.g., Merckx & Van Dyck, 2006). Interestingly, our results suggest that seasonal variation in wing size and shape is associated with contrasting flight characteristics (Table 2). This is in line with previous studies proposing that morphological differences among seasonal *Pieris* forms might be related to adaptive flight behaviours, with the spring forms being more sedentary and the summer form more dispersive (Fric et al., 2006; Shkurikhin & Oslina, 2016). In this regard, the elongated forewings of smaller spring *Pieris* compared to the summer forms (Fric et al., 2006; Shkurikhin & Oslina, 2016) were associated with a slower flight. Specifically, spring individuals exhibited low velocity, acceleration, advance ratio and turning acceleration, as well as a high flight curvature, which might indicate manoeuvrable flights with tortuous trajectories. Although our experiment provides a first insight into the differentiation of flight behaviours between seasonal butterfly forms, future experiments with captive‐bred individuals in controlled conditions are needed to ascertain whether the differences observed between seasonal forms might be adaptive.

Seasonal differentiation of butterfly adult morphology (size and shape) may be associated with divergent flight behaviours. These morpho‐behavioural changes may be explained by multiple non‐exclusive mechanisms, including larval development under different environmental conditions, selective pressures acting on flight, sexual selection or adaptive thermoregulation. For example, larval growth is affected by host plant species and their quality (Hwang et al., 2008), and by ambient temperatures and photoperiod (Nylin, 1994). Low ambient temperatures experienced by immatures lead to the development of larger adult individuals, which might have faster flights (Büyükyilmaz & Tseng, 2022), as also reported in our study for the *Pieris* summer form (Table 2). Further, it has been proposed that the shorter days during the autumn might trigger an earlier development of the overwintering stage, pupae, resulting in reduced size of adult individuals in the coming spring (Wiklund et al., 1991). In the light of our results, an alternative explanation is that seasonal difference in wing shape and size may be adaptive, resulting from distinct environmental pressures exerted on flight performance between seasons, as also suggested by Fric et al. (2006) and Shkurikhin and Oslina (2016). However, regardless of whether seasonal flight behaviour differences in *Pieris* are adaptive or plastic, their flight behaviours would also likely be affected by larval developmental and morphology disruptions driven by ongoing climatic changes (Moradinour et al., 2023).

Our approach revealed significant quantitative differences in flight behaviour between seasonal forms of *Pieris* (Table 2) and indicates an effect of wing morphology on flight (Table 3). However, using wild specimens, as in our study, likely results in uncontrolled variables such as variation in age, mating or feeding status, possibly impacting flight behaviours (Almbro & Kullberg, 2008, 2012). Similarly, we have not disentangled the potentially confounding effect of sex on flight behaviour (Van Dyck & Wiklund, 2002; Wiklund et al., 1991), due to our low sampling including only five females (Table 1). Further studies investigating the effects of varying environmental conditions (e.g. air temperature and host plant) during larval and adult stages on flight are needed to ascertain whether the seasonal flight divergence observed in *Pieris* butterflies is adaptive. In spite of these limitations, we discuss below the possible selective mechanisms at play that may drive seasonal flight divergence.

### 4.1 Seasonal but not interspecific morphological differences affect flight behaviour

Differences in environmental conditions between spring and summer may have driven divergent evolution of flight behaviours in *Pieris* butterflies (Table 2). While the two closely related species *P*. *napi* and *P*. *rapae* have variable, but overall undistinguishable flights (Table 2) and wing morphologies (Table 1), their respective seasonal forms appear to diverge in morphology (Table 1) and flight behaviour (Table 2). The observed morphological differences between seasonal forms are consistent with previous studies. For example, Fric et al. (2006) and Shkurikhin and Oslina (2016) hypothesised that the flight behaviour of spring individuals might be advantageous to explore resources on their natal patch, while the summer individuals might use a more dispersive flight behaviour to reach novel localities. Enhanced dispersal abilities during the summers have been proposed in other butterfly groups as well, as it may favour access to newly emerging larval feeding plants and possibly reduced parasitoid pressure (Kerr et al., 2020; Ohsaki & Sato, 1999).

Based on morphological differences and high wing loading, it was generally assumed that spring individuals would fly faster and with greater manoeuvrability than the summer individuals (Fric et al., 2006; Shkurikhin & Oslina, 2016). Contrary to such expectations, we found that the flight of spring *Pieris* was significantly slower than that of summer individuals (Table 2). A lower flight speed in smaller individuals of *P*. *rapae* – albeit showing higher wing loading – was also observed by Büyükyilmaz and Tseng (2022), who used a flight mill to measure velocity and covered distance of those butterflies. Similarly, Almbro and Kullberg (2012) noted that high wing loading is associated with reduced flight speed in *P*. *napi* males, but not in females. These findings suggest that seasonal differences in flight behaviour may not be solely conditioned by morphology. Indeed, determining the effect of morphology on performance is often challenging, as differences may only be apparent when individuals are pushed to their limits (Losos et al., 2002). Despite the general expectation that flight speed increases with wing loading (Le Roy et al., 2019), *Pieris* contradicts this trend (Table 1), suggesting that wing morphology may have even more intricate effects on butterfly flight behaviours than generally assumed (Srygley, 1999).

Although we did not reveal any significant effects of mean ambient air temperatures recorded from a nearby weather station on *Pieris* flight behaviour, we remain cautious of this finding as our experimental setup did not allow for a direct assessment of the effect of microhabitat temperature. Because body temperatures might affect flight performance (Tsuji et al., 1986), it was expected that flight duration would have been constrained by cool spring temperatures, leading butterflies to land more frequently to warm up in sunlight (Shkurikhin & Oslina, 2016). Nevertheless, although we have not measured flight behaviours in controlled thermal conditions, the recorded quantitative differences in flight explained by morphological variables among seasonal forms of *Pieris* are in agreement with previous evidence (e.g. Büyükyilmaz & Tseng, 2022).

The spring *Pieris* form was predicted to have more manoeuvrable flights compared to the straight flight of the dispersive summer form (Fric et al., 2006; Shkurikhin & Oslina, 2016). We provide quantitative evidence for this prediction, as suggested by a higher flight curvature of spring *Pieris* (Table 2), which was significantly associated with their elongated forewing shape (Table 3). The flight behaviour of spring *Pieris* might increase navigation and exploration efficiency in adult individuals living during early spring (Root & Kareiva, 1984) and be beneficial for locating emerging nectar sources and larval feeding plants (Danks, 2007). Although the benefits of adopting a curvy flight pattern for foraging in a dispersed resource environment remain hypothetical, the higher flight curvature of spring *Pieris* may have evolved jointly with a specialised foraging strategy. The divergent flight behaviour of spring *Pieris* might, thus, possibly be adaptive and linked to movements across the landscape, which might result in more effective exploitation of newly emerging feeding and oviposition resources (Cant et al., 2005).

Our study, however, has a strong sampling bias towards male individuals, preventing us from concluding on the adaptive nature of the flight differentiation. Indeed, our findings may be explained by sexual differences in flight behaviours (Almbro & Kullberg, 2012). Male and female butterflies often exhibit distinct flight patterns across the landscape due to differences in their resource utilisation strategies and habitat use (DeVries et al., 2010; Evans et al., 2020). Males and females of *Pieris* also differ in their wing morphologies (Almbro & Kullberg, 2012) and developmental rates (Wiklund et al., 1991), which might lead to differences in flight behaviour (Van Dyck & Wiklund, 2002). In addition, male *Pieris* are protandric (i.e. they emerge before females during a season; Wiklund et al., 1991), search for females by low patrolling flight above vegetation (Dennis, 1982) and can fly longer distances than females (Ducatez et al., 2012). Here we were not able to detect any sexual differences in flight behaviour likely because only five females were studied. Such a low sample is typically due to their lower flying abundances and/or more cryptic flight behaviour in nature compared to patrolling males. Future studies focusing on quantifying the flight behaviours of more female individuals may highlight sexual differences. Females are, for instance, expected to show straighter flights compared to patrolling males, and may exhibit flight differences stemming from fluctuations of body mass due to mating and oviposition (Almbro & Kullberg, 2012).

The summer dispersal of *Pieris* might facilitate the colonisation of novel patches with larval feeding plants and release from parasitoid pressure (Kerr et al., 2020; Ohsaki & Sato, 1999). Such dispersal ability was linked to the larger wing area of summer forms and, consequently, the lower wing loading characterising *Pieris* summer form (Fric et al., 2006; Shkurikhin & Oslina, 2016). It was further suggested that more rounded wings might be adaptive when using ascending thermal air currents for dispersal (Fric et al., 2006). Indeed, the larger forewing area typical of the summer form was significantly associated with high advance ratio, thus, potentially contributing to more effective dispersal (Table 3). Nevertheless, we did not find evidence supporting that the larger wing size of summer *Pieris* decreases acceleration, as predicted by Shkurikhin and Oslina (2016), and instead, we found higher acceleration and turning acceleration in summer *Pieris*. Such a powered flight may reflect the fast linear flight typically used during dispersive flight (Cant et al., 2005), although it is also known that dispersal is affected by warmer summer weather (Cormont et al., 2011). The higher acceleration and turning acceleration of the summer form might also represent seasonal behavioural responses to increased predator pressure on adult butterflies during summer, such as naïve juvenile birds (Zvereva & Kozlov, 2021) and dragonflies (Sang & Teder, 2011). This hypothesis should, however, be tested against predictions of higher predation pressure on insects during spring than during summer in Europe (e.g. in Scandinavia, Remmel et al., 2009) and probably also in Czechia. Although *Pieris* butterflies are palatable, free‐flying *Pieris* were also reported to be actively avoided by birds (Lyytinen et al., 1999). Thus, we argue that the faster flight with higher acceleration recorded in the summer forms could also represent an adaptive response in relation to predation.

Our results highlight the high intraspecific variability in butterfly flight behaviours (Figure 2), which challenge the constancy of simple biomechanics assumptions based on wing morphology alone (for a review, see Le Roy et al., 2019). Wing shape variation in temperate butterflies is typically plastic or adaptive, as revealed in *Pararge aegeria* (Nymphalidae) (Berwaerts et al., 2002; Berwaerts & Van Dyck, 2004; Van Dyck & Wiklund, 2002). Similarly, subtle sexual differences in wing shape are associated with distinct dispersal capacity in females *Melitaea cinxia* (Nymphalidae) (Breuker et al., 2007). Sexual dimorphism in wing size may also result from protandry (i.e. males emerge before females) and polyandry (females are mating with multiple males), as well as from trade‐offs between growth rate and emergence time (Wiklund et al., 1991). These alternative explanations emphasised that insect wing is a complex trait, evolving under the effect of multiple and sometimes conflictive selective pressures (Wootton, 1992). Our results add to mounting evidence that the predictors of flight behaviour such as manoeuvrability and dispersal capacity are complex and are likely the result of both morphological adaptation and behavioural intraspecific variation.

## 5 CONCLUSIONS

Our study reveals divergent flight behaviours between seasonal forms of two *Pieris* species. We provide quantitative evidence relating wing morphology, in particular the shape of forewings and forewing area, to differences in flight behaviour (Le Roy et al., 2019). We argue that different foraging flight behaviours (e.g. higher flight curvature of spring *Pieris* in relation to resource exploration) possibly promote the seasonal divergence in *Pieris* flight, a hypothesis that has rarely been tested in butterflies (e.g. Dell'Aglio et al., 2022; Mena et al., 2020; Spieth & Cordes, 2012). Future work should ideally combine laboratory experiments using reared individuals (Almbro & Kullberg, 2008) with flight recordings of wild butterflies in both controlled and natural settings (e.g. Cant et al., 2005; Maggiora et al., 2019) to clarify the mechanisms, whether adaptive or plastic, driving the seasonal differences in flight behaviour.

## AUTHOR CONTRIBUTIONS

**Irena Kleckova:** Conceptualization (equal); investigation (equal); methodology (equal); writing – original draft (lead); writing – review and editing (equal). **Daniel Linke:** Data curation (equal); formal analysis (equal); methodology (equal); writing – review and editing (equal). **Francisko De Moraes Rezende:** Conceptualization (equal); investigation (equal); writing – review and editing (equal). **Luca Rauscher:** Data curation (equal); formal analysis (equal); writing – review and editing (equal). **Camille Le Roy:** Data curation (equal); formal analysis (equal); methodology (equal); writing – review and editing (equal). **Pável Matos‐Maraví:** Conceptualization (equal); funding acquisition (equal); investigation (equal); methodology (equal); supervision (equal); writing – review and editing (equal).

## FUNDING INFORMATION

This work was supported by the Czech Science Foundation, Junior GAČR grant GJ20‐18566Y to P.M.M. and by the PPLZ program of the Czech Academy of Sciences (fellowship grant L20096195 to P.M.M.) as well as by the Grant Agency of the University of South Bohemia, GAJU n. 014/2022/P to D.L. and F.M.R.

## CONFLICT OF INTEREST STATEMENT

We declare no competing interests.

## Supporting information


Data S1:

## Data Availability

All data and scripts are archived in the Figshare repository (DOI:https://doi.org/10.6084/m9.figshare.24631410.v6).
