# Supplementary material for: Flight behaviour diverges more between seasonal forms than between species in Pieris butterflies
Source: Ecol Evol. 2024 Jul 17;14(7):e70012. doi: 10.1002/ece3.70012 (PMC11255373; doi:10.1002/ece3.70012)

SUPPLEMENTARY MATERIALS

Title: Flight behaviour diverges more between seasonal forms than between species in *Pieris* butterflies

Running title: seasonal differences in flight behaviour of *Pieris* butterflies

Authors:

Irena Kleckova[1*], Daniel Linke[1,2], Francisko De Moraes Rezende[1], Luca Rauscher[2], Camille Le Roy[3], Pável Matos-Maraví[1*]

Affiliations:

[1] Institute of Entomology, Biology Centre CAS (Czech Academy of Sciences), České Budějovice, Czechia

[2] Department of Zoology, Faculty of Science, University of South Bohemia, České Budějovice, Czechia

[3] Experimental Zoology Group, Wageningen University, Wageningen, the Netherlands

*Correspondence authors: Irena Kleckova, e-mail: irena.slamova@gmail.com; Pável Matos-Maraví, e-mail: pavel.matos@entu.cas.cz

Key words

Flight kinematics, trajectory tracking, Lepidoptera, morphometrics, polymorphism

Table S1. Pearson correlation coefficients among various morphological variables describing the forewing size and shape of *Pieris* butterflies. Forewing area correlates with aspect ratio, while other morphological variables were not correlated to each other.

		Correlation coefficient	P_value
**Forewing area**	**Aspect ratio**	**-0.40**	**0.03**
Forewing area	Wing loading	-0.10	0.60
Forewing area	Forewing edge	-0.28	0.14
Forewing area	Marginal region	-0.20	0.29
Aspect ratio	Wing loading	0.18	0.34
Aspect ratio	Forewing edge	0.31	0.09
Aspect ratio	Marginal region	0.36	0.05
Wing loading	Forewing edge	-0.04	0.82
Wing loading	Marginal region	0.13	0.49
Forewing edge	Marginal region	0.14	0.45

Table S2. Mean ambient temperatures for each recording day do not correlate with flight characteristics of *Pieris napi* and *P. rapae*. Butterfly species did not differ in flight behaviour, but their seasonal forms do. The linear mixed models (LMMs) include individual identity as a random factor and log-transformed covered distance and sex as fixed factor. Significant relationships are highlighted **in bold**.

Flight characteristic	Variable	Sum Sq	Mean Sq	NumDF	DenDF	F value	Pr(>F)
Wingbeat frequency	Tmean_recoding_hours	0.00	0.00	1.00	29.55	0.00	0.99
	species	0.02	0.02	1.00	29.78	0.27	0.60
	season	0.04	0.04	1.00	30.33	0.47	0.50
Flight height	Tmean_recoding_hours	0.00	0.00	1.00	28.75	0.33	0.57
	species	0.00	0.00	1.00	29.65	0.00	0.96
	season	0.03	0.03	1.00	30.32	2.06	0.16
**Velocity**	Tmean_recoding_hours	0.12	0.12	1.00	27.54	0.83	0.37
	species	0.00	0.00	1.00	28.34	0.01	0.94
	**season**	**1.26**	**1.26**	**1.00**	**28.92**	**8.65**	**0.01**
**Advance ratio**	Tmean_recoding_hours	0.24	0.24	1.00	29.84	1.11	0.30
	species	0.13	0.13	1.00	29.45	0.59	0.45
	**season**	**1.30**	**1.30**	**1.00**	**29.34**	**5.90**	**0.02**
**Acceleration**	Tmean_recoding_hours	0.03	0.03	1.00	27.50	0.12	0.73
	species	0.00	0.00	1.00	28.36	0.01	0.92
	**season**	**1.29**	**1.29**	**1.00**	**28.99**	**5.28**	**0.03**
**Turning acceleration**	Tmean_recoding_hours	0.04	0.04	1.00	27.55	0.11	0.74
	species	0.02	0.02	1.00	28.62	0.06	0.81
	**season**	**3.25**	**3.25**	**1.00**	**29.44**	**9.46**	**0.00**
Turning rate	Tmean_recoding_hours	0.10	0.10	1.00	26.21	0.40	0.53
	species	0.01	0.01	1.00	27.88	0.05	0.83
	season	0.03	0.03	1.00	29.30	0.12	0.73
**Curvature**	Tmean_recoding_hours	0.37	0.37	1.00	29.48	0.54	0.47
	species	0.08	0.08	1.00	31.03	0.11	0.74
	**season**	**10.34**	**10.34**	**1.00**	**32.28**	**14.97**	**0.00**
Ascent angle	Tmean_recoding_hours	0.36	0.36	1.00	21.50	1.90	0.18
	species	0.00	0.00	1.00	25.66	0.00	0.98
	season	0.16	0.16	1.00	29.82	0.85	0.36
Sinuosity	Tmean_recoding_hours	0.00	0.00	1.00	30.90	0.03	0.86
	species	0.07	0.07	1.00	32.99	1.26	0.27
	season	0.02	0.02	1.00	34.82	0.36	0.55

Supplementary figure 1.

Importance of each morphological predictor, and sex and covered distance (fixed effects) revealed by a dredge analysis using the R package MuMIn (Barton 2009) followed by per-variable sum of model weights analyses. The algorithm considers all possible combinations of predictors to evaluate and rank a comprehensive set of candidate models that can be formed from a global model.

(1) Wingbeat frequency

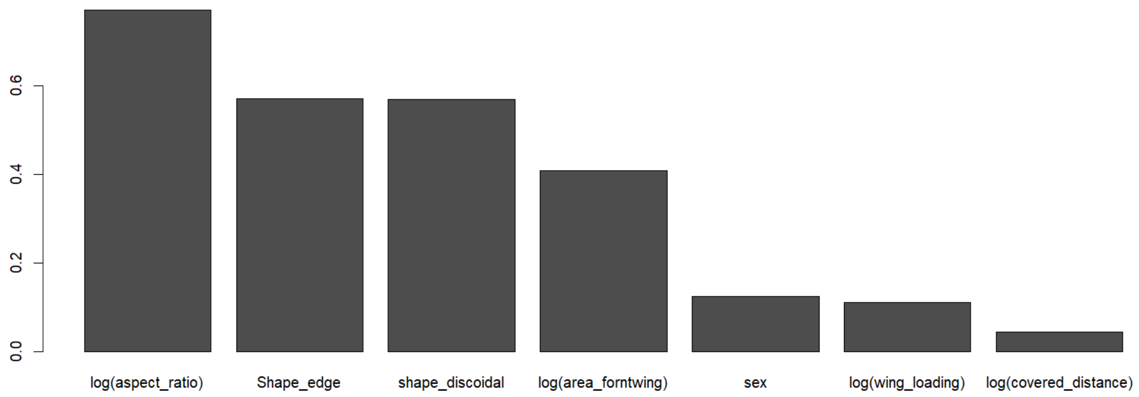

(3) Flight height

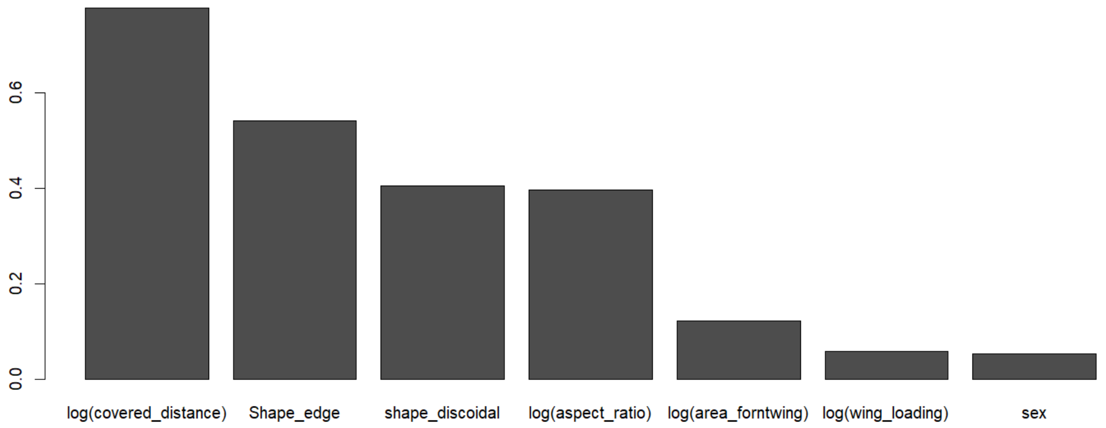

(4) Velocity

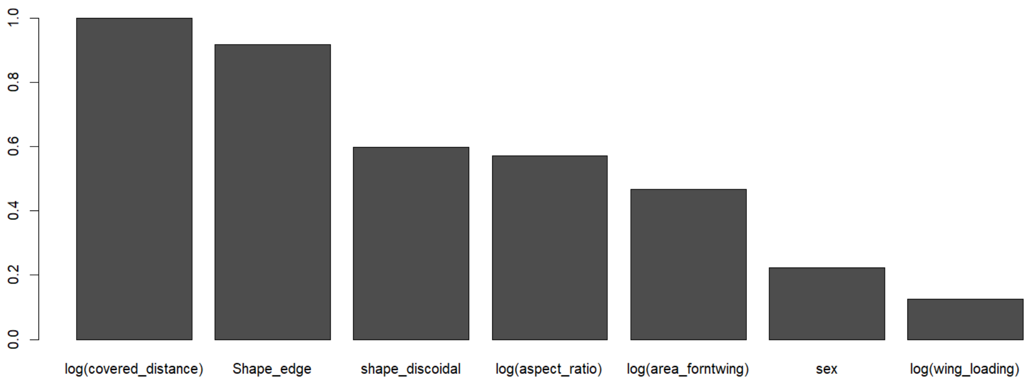

(5) Acceleration

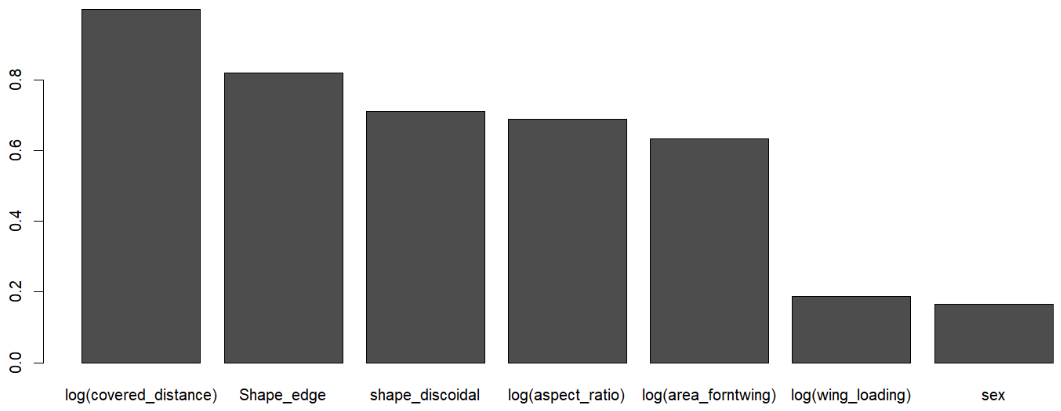

(6) Advance ratio

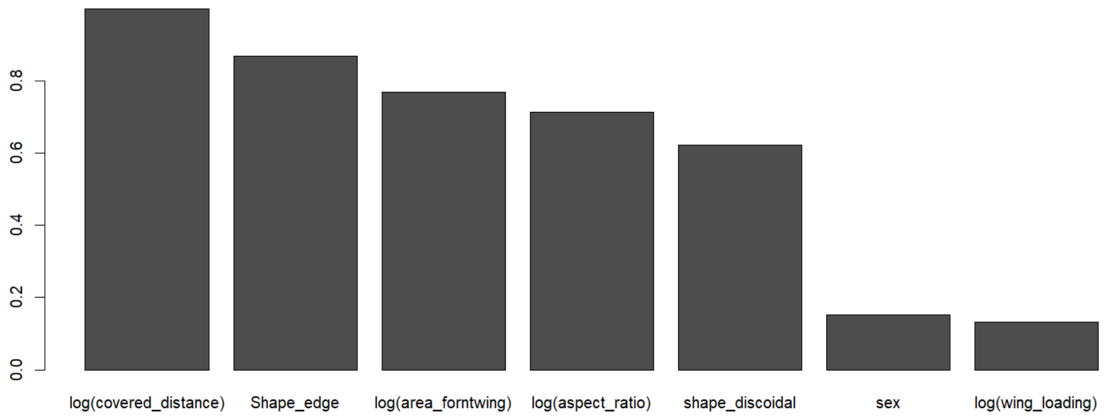

(7) Turning acceleration

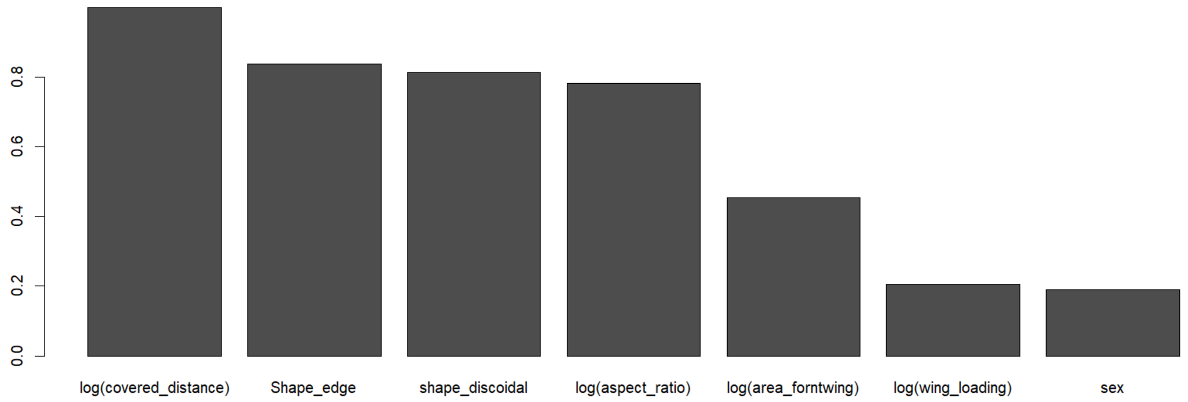

(8) Turning rate

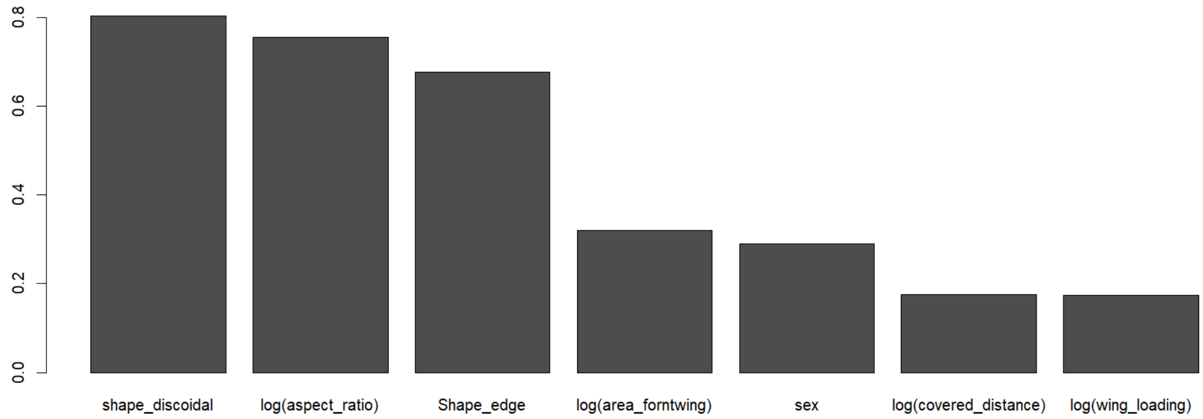

(9) Sinuosity

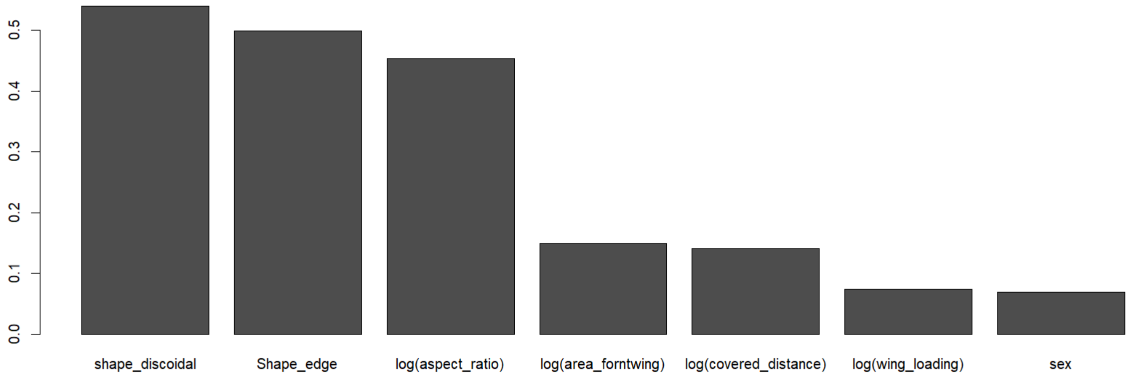

(10) Curvature

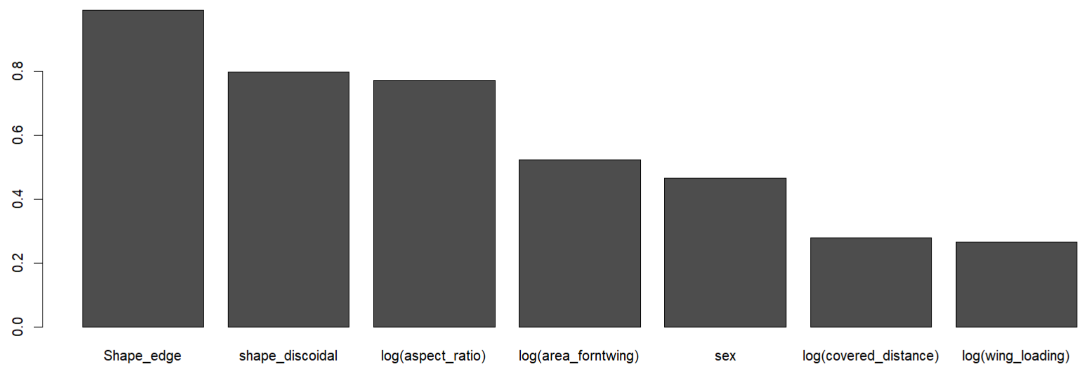

(11) Ascent angle

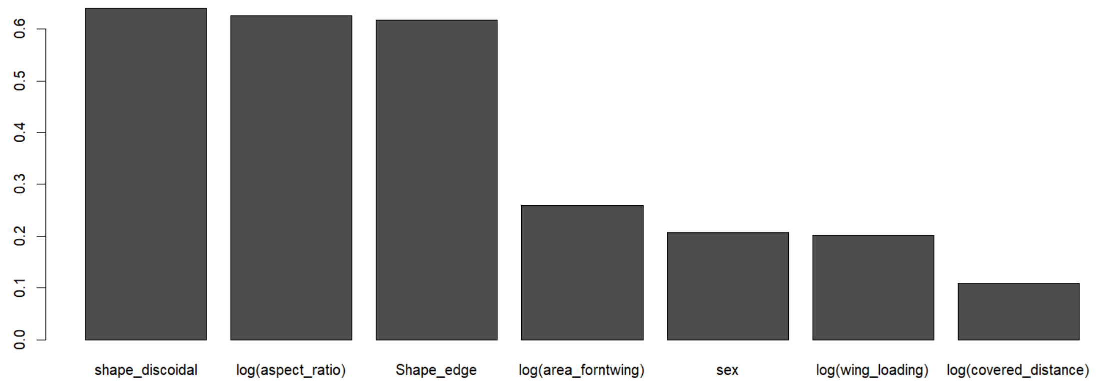

Supplement: Supplementary file 1 — Data S1: [file ECE3-14-e70012-s001.pdf]
